# Melanopsin-Derived Visual Responses under Light Adapted Conditions in the Mouse dLGN

**DOI:** 10.1371/journal.pone.0123424

**Published:** 2015-03-30

**Authors:** Katherine E. Davis, Cyril G. Eleftheriou, Annette E. Allen, Christopher A. Procyk, Robert J. Lucas

**Affiliations:** Faculty of Life Sciences, University of Manchester, Manchester, United Kingdom; University College London, UNITED KINGDOM

## Abstract

A direct projection from melanopsin-expressing intrinsically photosensitive retinal ganglion cells (ipRGCs) reaches the primary visual thalamus (dorsal lateral geniculate nucleus; dLGN). The significance of this melanopsin input to the visual system is only recently being investigated. One unresolved question is the degree to which neurons in the dLGN could use melanopsin to track dynamic changes in light intensity under light adapted conditions. Here we set out to address this question. We were able to present full field steps visible only to melanopsin by switching between rod-isoluminant ‘yellow’ and ‘blue’ lights in a mouse lacking cone function (Cnga3^-/-^). In the retina these stimuli elicited melanopsin-like responses from a subset of ganglion cells. When presented to anaesthetised mice, we found that ~25-30% of visually responsive neurones in the contralateral dLGN responded to these melanopsin-isolating steps with small increases in firing rate. Such responses could be elicited even with fairly modest increases in effective irradiance (32% Michelson contrast for melanopsin). These melanopsin-driven responses were apparent at bright backgrounds (corresponding to twilight-daylight conditions), but their threshold irradiance was strongly dependent upon prior light exposure when stimuli were superimposed on a spectrally neutral ramping background light. While both onset and offset latencies were long for melanopsin-derived responses compared to those evoked by rods, there was great variability in these parameters with some cells responding to melanopsin steps in <1 s. These data indicate that a subset of dLGN units can employ melanopsin signals to detect modest changes in irradiance under photopic conditions.

## Introduction

Photoreception in the mammalian retina is not restricted to rods and cones but extends to a population of intrinsically photosensitive retinal ganglion cells (ipRGCs) expressing the photopigment melanopsin [1, 2]. Accounting for <5% of the ganglion cell population, ipRGCs are known to encode ambient light levels (irradiance) for circadian and pupillary reflex systems [1, 3, 4]. However, ipRGCs also project to the dLGN [5–7] implying a contribution to more conventional aspects of visual discrimination.

The role of ipRGCs in the primary visual pathway is only gradually being elucidated: recordings from dLGN neurons in mice confirm that a substantial fraction are influenced by melanopsin photoreceptors, enhancing their ability to encode the irradiance of steps from darkness [5]; psychophysical studies in humans indicate that melanopsin can support brightness discrimination [8, 9]; behavioural evidence from retinally-degenerate mice shows that melanopsin alone is sufficient to use crude visual cues for maze navigation [8]; while melanopsin knockout mice display deficits in several aspects of visual function and development [10–13].

To date, most studies examining ipRGC responses have employed pulses of light on a background of darkness, in effect assessing ipRGC function under dark-adapted conditions. To begin to understand melanopsin’s contribution to pattern vision a description of the sensory capabilities of melanopsin-driven responses in dLGN under more naturalistic light-adapted states is required. Over what range of background light intensities does melanopsin influence dLGN firing? Does the dLGN use melanopsin to merely track background light intensity or can it encode more dynamic changes in lighting? How sensitive is the melanopsin signal to changes in light intensity?

Addressing these questions requires a method of isolating visual responses driven by melanopsin from those elicited by rods and/or cones. Here, we first undertake *in vitro* retinal recordings to explore two potential solutions to this problem: 1.) using an established transgenic line lacking rods and cones (*rd/rd cl*); and 2.) using receptor silent substitution to selectively modulate melanopsin excitation. As the technically and conceptually simpler of the two, we started by recording responses in the *rd/rd cl* retina under light adapted conditions. We found a number of units responding to light increments, with increases in firing that were very slow to build up and dissipate, and with poor overall reproducibility. For the silent substitution approach we have previously separated melanopsin from cone-driven responses at high (rod-saturating) background lights in visually-intact animals [8]. However, separating melanopsin from rod-driven responses at mesopic irradiances is complicated by similarities in the spectral sensitivity of these two receptors. Here we adopted a compromise: we used Cnga3^-/-^ mice which retain rod function and whose inner retinal circuitry is anatomically intact, but in which cone photoreceptors are rendered non-functional (locked in a hyperpolarized state equivalent to a continuous light response [14]). Our strategy was then to isolate melanopsin-based responses in this model by switching between ‘blue’ and ‘yellow’ lights whose intensity was set to make them indistinguishable for the remaining outer retinal photoreceptors (rods). We found that these melanopsin-isolating stimuli successfully elicited responses from a small population of neurones in the retinal ganglion cell layer that were faster and more repeatable than those recorded from the *rd/rd cl* retina but much slower than expected if they were elicited by rods. Such responses were retained following pharmacological blockade of synaptic input but were less robust and had poorer kinetics, consistent with the view that melanopsin-responses were assisted by the partially intact retinal circuitry. Given the higher quality of responses achieved with the silent substitution approach, we applied this to record melanopsin-evoked activity in the dLGN. We found that, under these light adapted conditions, melanopsin-driven responses in the dLGN could be recorded over a range of bright background irradiances, but appeared strongly influenced by light adaptation. Under the right conditions even fairly modest changes in effective melanopsin irradiance (32% Michelson contrast; ~x2 increase) elicited measurable responses.

## Methods

### Ethics

The care and use of all mice in this study was carried out in strict accordance with UK Home Office regulations, UK Animals (Scientific Procedures) Act of 1986 (revised in 2012) and approved by the local Manchester Animal Welfare and Ethical Review Board (AWERB reference 50/02506). All *in vivo* surgical procedures were performed under terminal urethane anaesthesia. Animals used for *in vitro* recordings were killed by Schedule 1 cervical dislocation by fully trained personnel. In both cases all efforts were made to minimise suffering.

### Animals

Recordings were made on male Cnga3^-/-^ ‘coneless’ mice of between 50 and 120 days of age and male *rd/rd cl* ‘rodless and coneless’ mice of approximately 200–220 days. Mice were bred at the in house SPF facility at the University of Manchester and kept on a 12:12 light/dark cycle with ad libitum food and water.

### Retinal recordings

Following a period of dark-adaptation (one hour), mice were killed by cervical dislocation, eyes removed and left submerged in carboxygenated (95% O2 / 5%CO2) aCSF (artificial cerebro-spinal fluid, concentration in mM: 118 NaCl, 25 NaHCO_3_, 1 NaH_2_PO_4_, 3 KCl, 1 MgCl_2_, 2 CaCl_2_, 10 D-glucose, 0.5 L-Glutamine) throughout the dissection. Immediately following enucleation, the eyes were incised and vitreous humour and lens removed (all procedures occurring under dim red light). Eyecups were kept for two hours in 200 ml of carboxygenated aCSF in complete darkness. Thereafter the retina was removed from the eyecup using two pairs of very fine forceps and the remaining vitreous humour was gently removed. Care was taken to maintain as much RPE interdigitated with rod outer segments as possible. The retina was then mounted onto a 60 or 256 channel Multi Electrode Array (60MEA200/30iR-ITO or 256MEA200/30iR-ITO; GmbH, MultichannelSystems) with the ganglion cell layer facing down onto the electrodes and the optic disc to the side. Electrode contacts were 30 μm in diameter and were spaced at 200 μm pitch on a square grid. To improve cell contact coupling, a polyester membrane filter (5μm pores) held the retina in place whilst being weighed down by a stainless steel anchor (~0.75g) bearing a framework of parallel glass capillaries. The retina was left for 1 to 1.5 hours on the array to settle and dark-adapt prior to commencing recordings. Electrophysiological signals were acquired using MC_Rack software (Multi Channel Systems, Reutlingen, Germany) through an MEA1060INV (for 60 channel recordings) or a USB-MEA256 amplifier (for 256 channel recordings; Multi Channel Systems, Reutlingen, Germany) via a USB-MC acquisition card. Recordings were made at 25 kHz sampling frequency during the acquisition of both spontaneous and evoked activity. To preserve physiological conditions, the tissue was perfused with carboxygenated aCSF at 2.2 ml/minute using a peristaltic pump (SC120U, Watson Marlow, UK). To confirm the presence of ipRGCs, rod responses were abolished in some Cgna3^-/-^ experiments by applying a cocktail of drugs (75 uM L-AP4 and 40 uM NBQX) to the aCSF targeting glutamatergic synapses with bipolar cells. During the recording of electrophysiological activity, retinal explants were maintained at 32°C using a temperature controller (TC02, Multi Channel Systems, Reutlingen, Germany) regulating an inline heater for the inflow of aCSF (Ph01, Multi Channel Systems, Reutlingen, Germany).

### dLGN recordings

Mice were anaesthetised using a single dose of urethane (30% w/v in dH_2_O, 1.6mg/kg, i.p) and monitored until areflexia was achieved, upon which a tracheotomy was performed to open the airway and aid breathing. Where reflexes remained, a small top-up dose of urethane (10% w/v in dH_2_O, 20–30μl) was given. The mouse was placed in a stereotaxic frame (SR 5-M; Narishige, Japan) and core body temperature was maintained at 37oC on a homoeothermic heating blanket (Harvard Apparatus, UK). A midline scalp incision was made, and a square craniotomy was drilled in the right hemisphere above dLGN (B–2.2mm to 2.6mm, ML 1.5-3mm) relative to the coordinates of the mouse stereotaxic atlas [15]. A recording electrode (A4x8-5mm-50-200-177/413-A32; Neuronexus, USA) with 4 shanks and 8 contacts (50μm spacing) on each shank was lowered 2.7mm deep into dorsal LGN, providing a recording grid covering approximately 400 by 800μm. Extracellular spiking activity was collected through a Recorder64 system (Plexon, USA) and amplified through an AC coupled headstage x 20 (Plexon, USA) and by the system to give a total gain of 3500x. Signals were high-pass filtered (300Hz) and sampled at 40kHz. A mydriatic agent (Atropine 1%; Sigma, UK) was applied to the eye contralateral to the surgery site to dilate the pupil and mineral oil was applied to retain corneal moisture. A light cable, fitted with a lambersion diffuser (Edmund Optics) to deliver spectrally-uniform, full-field stimuli, was positioned at a distance of approximately 2mm. The ipsilateral eye was closed with tape to prevent binocular responses converging on contralateral dLGN. To aid electrode placement verification in post-hoc histology, electrodes were dipped in fluorescent dye (CM-Dil, Life Technologies, UK) prior to insertion and lesions were created at the four outmost contacts [16] at the end of the experiment. Mice were killed via cervical dislocation and brains were extracted and fixed in 4% paraformaldehyde. Histology for identification of electrode location was performed as described previously [17].

### Light stimuli

**Fig 1:**
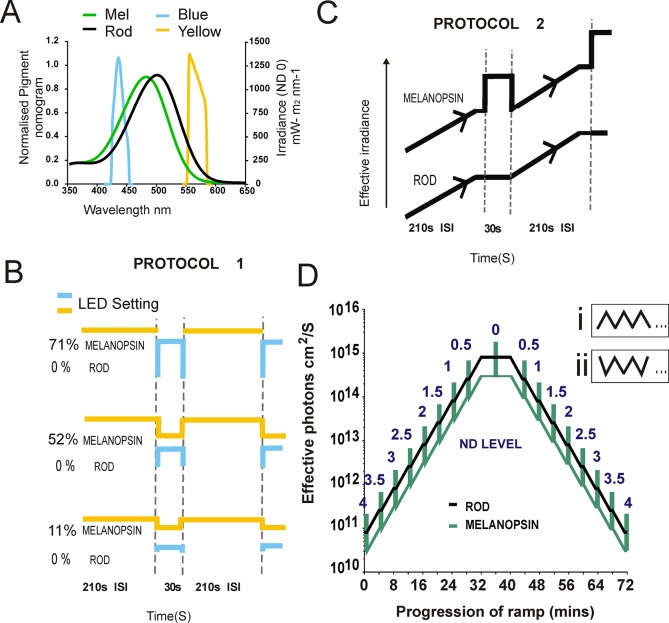
Light Stimulation Protocols. A: LED spectral power densities and *in vivo* photoreceptor spectral sensitivity (normalised). The output of blue and yellow LEDs was adjusted to produce equivalent effects on rods (black line). By contrast, the blue LED, always appeared brighter for melanopsin (green line). B: Protocol 1. Stimuli (30 or 20s melanopsin-isolating steps in dLGN and retina, respectively) presentations of the blue LED were interleaved with 210 or 180 sec of the (dLGN and retina, respectively) yellow to produce a ‘step’ visible only to melanopsin. The magnitude of this melanopsin step could be varied by mixing blue and yellow in the step (increasing the yellow and decreasing the blue elicited decreasing levels of contrast). C: Protocol 2. Starting at ND4, irradiance slowly ramped up (0.5 ND per 200 seconds) before remaining at a steady state for 10 seconds. At each 0.5ND, a blue melanopsin-isolating step (71%) is given for 30 seconds (total time per 0.5ND cycle = 4 minutes). This process was repeated until reaching ND0, at which point light-levels instead slowly ramped down and the process was repeated. D: The effective change in photon flux for melanopsin (green) and rods (black) across a full repeat of Protocol 2. Settings of ND filter at the point of each melanopsin isolating step are provided above. Di and ii: The starting position of the ramp (ND4 or ND0) was varied across experiments.

Our melanopsin-isolating stimuli consisted of a switch between yellow (‘background’; bg) and blue (‘step’) LEDs (λmax 575 and 438 respectively; Fig 1A) delivered by a Spectra X light engine (Lumencor, USA) and presented to the eye as diffuse illumination of a Lambertian disc (10 mm in diameter, placed <5mm from corneal surface). In the case of retina recordings, light stimuli were delivered via an optic cable fitted to the underside of the microscope stage holding the MEA-mounted retina.

Spectral power densities for each LED were measured using a calibrated spectroradiometer (Bentham Instruments Ltd., UK). These were converted to effective photon flux for rod opsin and melanopsin by multiplying by the normalised *in vivo* spectral sensitivity for each photopigment, correcting for the pre-receptoral filtering (in the case of *in vivo* recordings) and integrating across the spectrum ([18] spectral sensitivity functions available at http://lucasgroup.lab.ls.manchester.ac.uk/research/measuringmelanopicilluminance/). Based upon these calculations it was possible to generate stimulus pairs presenting equal effective photon flux for rod opsin, while differing in effective photon flux for melanopsin by up to 5.7 fold (71% Michelson contrast for melanopsin calculated using the equation: (step irradiance—background irradiance) / (step irradiance + background irradiance). To generate a range of melanopsin-isolating contrasts (71, 60, 51, 38, 32, 21 and 11%) the ‘step’ component contained increasing levels of yellow LED while decreasing the blue component (Fig 1B). In addition, a circular neutral-density (ND) wedge ((100FS04DV.4, Newport) controlled by NSC200 controller system, NewStep, USA) was introduced in the light path between the exit point of the light engine and the end of the optical fibre. This enabled us to deliver background stimuli over a 4 log unit range of light (3.08 x 10^10^ to 1.95 x 10^14^ photons/cm^2^/s for melanopsin; ND4-ND0, hereafter referred to as log photons/cm^2^/s or as ND levels), covering the mesopic to photopic range. Stimuli for retinal data were generated by the same method as for dLGN, with the exception that the LED outputs were measured through the microscope stage and estimates of photoreceptor spectral sensitivity did not include the pre-receptoral filtering correction. In order to facilitate comparisons between *in vivo* and *in vitro* data, in both cases we report the effective corneal irradiance throughout this manuscript. In the case of retinal recordings we used the method of Lyubarsky et al., [19] to convert measured retinal to predicted effective corneal irradiance by multiplying by pupil area/retinal area; a factor of 0.5 for a fully dilated pupil.

### Stimulation Protocols

Responses of the *rd/rd cl* retina to light steps were assessed using multielectrode array recordings. The output of the blue LED was modulated between a background and a step (12.8 and 13.5 log melanopsin-effective photons/cm^2^/s respectively; 71% effective contrast; interstimulus interval 180s; pulse duration 20 or 60s).

Both *in vivo* and *in vitro* preparations, experiments with Cnga3^-/-^ mice started with a definition of basic light response characteristics by recording responses to a rod-activating flash (50ms of the yellow LED attenuated by 10^-3 with the neutral density wedge (ND3; 10.9 log rod opsin-effective photons/cm^2^/s) at 1Hz) and a stimulus predicted to target both rods and melanopsin (10 sec blue pulses over a range of irradiances (11.21 to 15.1 log melanopsin-effective photons/cm^2^/s) separated by 50–110 sec darkness). For dLGN recordings, we additionally confirmed that the units from which we were recording had a relatively central receptive field by recording responses to a series of flashing white bars (8 horizontal and 8 vertical) displayed for 200ms at a time in sequence on a black LED TV screen (AOC e2070Swn 19.5” LED monitor), placed approximately 15cm from the mouse’s eye and covering 118 x 90 degrees of vision. The electrode was moved if there were few electrode contacts with a central receptive field in reference to the azimuth (centre section of screen covering 59 by 45 degrees of vision; [20]). For both Cgna3^-/-^ retinal and dLGN recordings, we included a phase of online calibration in which we identified the setting of a blue (step) stimulus that failed to elicit a measurable response from rods when matched with a yellow background (effective rod isoluminance; See results section). This was achieved by presenting a fast flash of the blue LED alternating with the yellow background (50ms blue:200ms yellow or 100ms blue:400ms yellow) at a moderate background light against which we could cleanly detect rod responses (ND3 = 11.92 and 11.45 log rod-opsin effective photons/cm^2^/s in dLGN and retina, respectively). By decreasing and increasing the intensity of the blue LED input around our calculated ‘isoluminant’ setting we could generate clear rod flash responses either side of our estimated point of rod isoluminance (see results). In almost all cases, we also identified a blue setting at which responses were absent, and this point of effective rod isoluminance was at or very close to our a priori estimate. A very small number of cells (10 of 375 from 15 Cgna3^-/-^ mice) retained responses under all settings of the blue that lay close to our prior estimate and were excluded from further analysis. We also re-tested these settings under bright light conditions (ND0 = log 14.91 and 14.3 log rod-opsin effective photons/cm^2^/s in dLGN and retina, respectively) to ensure it was rod silent before commencing with the main protocols. Finally, in order to confirm that the results of this online calibration flicker test were sufficient to detect rod responses to small levels of contrast, we estimated the rod contrast sensitivity for fast flicker and sustained steps in a subset of preparations (4 *in vivo* and 3 *in vitro*). In brief we calculated melanopsin-isoluminant blue and yellow stimuli and presented transitions between a blue background and yellow flash as either 4Hz flicker (50ms:200 ms of bg) or 30 sec step and over a range of estimated contrasts for rods (4, 15 and 30% rod contrast) in order to a lower bound for the effective rod contrast detectable with these protocols.

Having defined effective rod isoluminant stimulus pairs for each recording online we presented the melanopsin-isolating step against either a steady (protocol 1) or changing (protocol 2) background irradiance (Fig 1B and 1C). To start, the retina/mouse (n = 2 and 5) was held for 10 mins under a bright background (yellow LED; retina 12.75 log melanopsin-effective photons/cm^2^/s and *in vivo* recordings 14.3 log melanopsin-effective photons/cm^2^/s) after which a series of 20 sec (30 sec for *in vivo*) melanopsin steps were delivered. In the case of *in vitro* retinal recordings all stimuli were presented at a 71% melanopsin contrast only. In these experiments, the melanopsin-isolating stimuli was first presented for 10 repeats under normal aCSF conditions and following the application of synaptic blockers (blockade was confirmed upon the loss of a rod-favouring flash response within 5 minutes of perfusion), repeated a further 10 times, enabling us to view the synaptically-isolated ipRGC response. Difficulties associated with injecting synaptic blockers into the eye while electrodes were in place and the animal was in the stereotaxic frame precluded this approach from *in vivo* recordings. For *in vivo* recordings, steps at 4 differing melanopsin contrasts (71–11%) were interleaved pseudo-randomly for at least 14 repeats at an ISI of 210 seconds. In a separate set of recordings (10 for dLGN and 8 for isolated retinas), we delivered a set of 71% melanopsin contrast steps over a 4 log unit range of background light intensities (Protocol 2; Fig 1C and 1D). In brief, the protocol started with 4 minutes of the yellow background at ND4 (dLGN; 10.5 log melanopsin-effective photons/cm^2^/s) after which a single 30 sec melanopsin-isolating step was presented. Upon termination of this step, the neutral density wedge was used to gradually increase the yellow background by half a log unit over 200 seconds. This new background (ND 3.5) was held for 10 sec before presenting the melanopsin-isolating step again. This process was repeated until the highest background was reached (ND0; dLGN 14.3 log melanopsin-effective photons/cm^2^/s) upon which the protocol was repeated for gradually decreasing background irradiance (See Fig 1D for ramping irradiance in log photons for dLGN). It is important to note that during the ramping stage of the stimulus the relative photons for both rods and melanopsin increased, but during the 30 sec step, only melanopsin would have been able to detect the change. The total time for a ramp up and down was 72 minutes, with an additional period of 4 minutes of light adaptation to ND4 at either end. This protocol was repeated 3 or 4 times for each preparation. For 4 experiments of each (dLGN and retina), instead of starting the first repeat ramping upwards from ND4, we started from ND0 and ramped down for the first presentation (Fig 1Dii). As there were no obvious difference in responses under these two conditions, data for the separate arms were pooled for analysis. For 2/8 of the experiments conducted in retina we also examined the activity of the ramp when under synaptic blockade.

### Data analysis

Data from dLGN and isolated retina were analysed in the same way: single-units were isolated from the multi-unit activity by sorting spike waveforms on the basis of principle component analysis and spike rate interval (Offline Sorter v3, Plexon, USA). Data in the form of trial bin count plots, peri-event histograms (PSTH), rate histograms, raster plots and absolute firing rates were analysed for each single unit using a combination of NeuroExplorer (v4; Plexon, USA) and a series of custom-built programmes in matlab (R2013a, The Mathworks Inc, UK). Group data was then further analysed statistically using Prism (v6; GraphPad). In all cases, cells were first identified as being light responsive by comparing the baseline firing rate to that following the flash (or during the step) using a student’s t-test. Cells responding to the melanopsin isolating stimuli where then further compared (factor 1; baseline vs step firing rate, factor 2 irradiance, step contrast or drug condition) with repeated measures ANOVA and Bonferoni post-hoc comparisons. Data are presented in graphs as either the mean peak firing rate (firing rate spikes/sec over the 30 sec step period) or Δ firing rate (mean peak firing rate subtracted from the mean baseline 10/30 sec prior to stimulus) and in some TBC plots as a normalised firing rate, where responses were scaled to the maximum firing rate over the presented epoch to account for changes in baseline activity over the duration of the protocol. Onset and offset latencies, presented in scatter plots were calculated as the time after stimulus onset/offset respectively at which firing rate crossed the line of 1 standard deviation above mean baseline after stimulus onset and offset. Receptive field plots was created according to the methods of Allen et al ([20]; data not shown).

## Results

### Melanopsin-driven responses under light adapted conditions in the rodless+coneless retina

**Fig 2:**
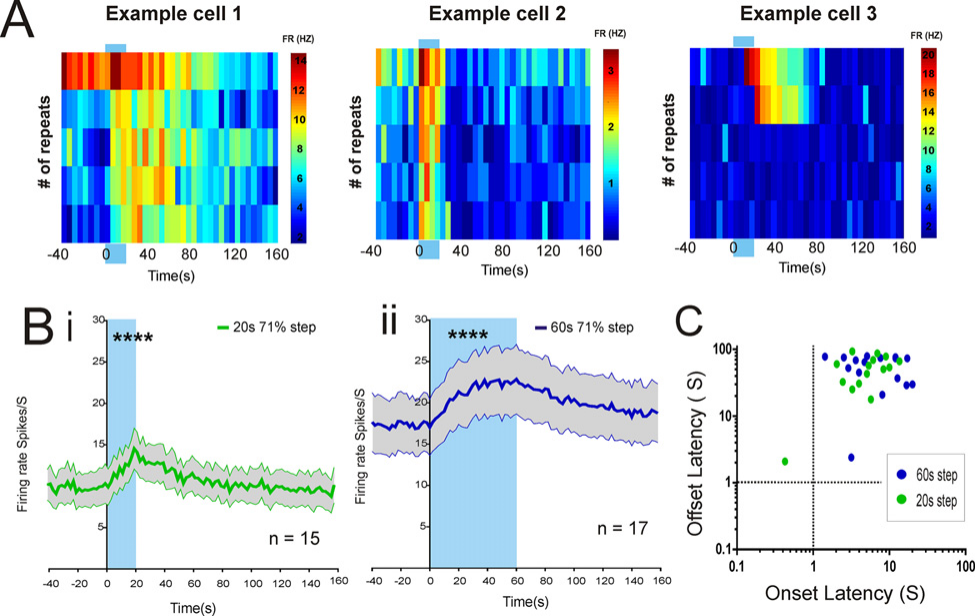
Retinal responses in *rd/rd cl* mice. A: Trial bin count examples (5 second bins of time) of three cells responding to a 20s 71% contrast step (background log 12.8 to step log 13.56 melanopsin photons/cm2/s) in a *rd/rd cl* retina. Note the variety in the latency of response offset in examples 1 and 2, and the poor response reproducibility that is typical of *rd/rd cl* mice in example 3. Colour bar to the right of the plot (FR Hz) denotes the firing rate of cells in this and subsequent Trial bin count figures. B: Averaged plots for firing rate over time of melanopsin-step responsive cells (mean±SEM) to a 20s step (Bi n = 15) and a 60s step (Bii n = 17). Both step durations elicit a significant change in firing to the baseline rate (**** P<0.0001). C: A plot of mean onset and offset latencies for each individual responsive cell over multiple stimulus repeats reveals the extremely sluggish nature of light responses in *rd/rd cl* mice.

In 3 recordings of *rd/rd cl* retinas presented with 20s steps, and 1 recording with 60s 71% contrast steps (256 recording sites each), we found 32 units with a significant stimulus-associated change in firing (Fig 2A and 2B; paired t-tests t(14) = 6.95, P<0.0001) for 20s and t(16) = 5.74, P<0.0001 for 60s). In all cases, the step induced an increase in firing rate, but units differed substantially in the reliability of this response, with some units responding only on a minority of stimulus repeats. In most cases, response onset latency lay between 1 and 10s after the start of the step, while in all but two cases offset latency was >10s (Fig 2C; mean+/SEM onset 5.72 +/- 0.89, and offset 50.96 +/- 6.69, n = 15 for 20s steps; onset 8.11s +/- 1.43 and offset 55.69 +/- 5.8, n = 17 for 60s steps).

### Light-adapted Melanopsin-driven responses in the coneless retina

**Fig 3:**
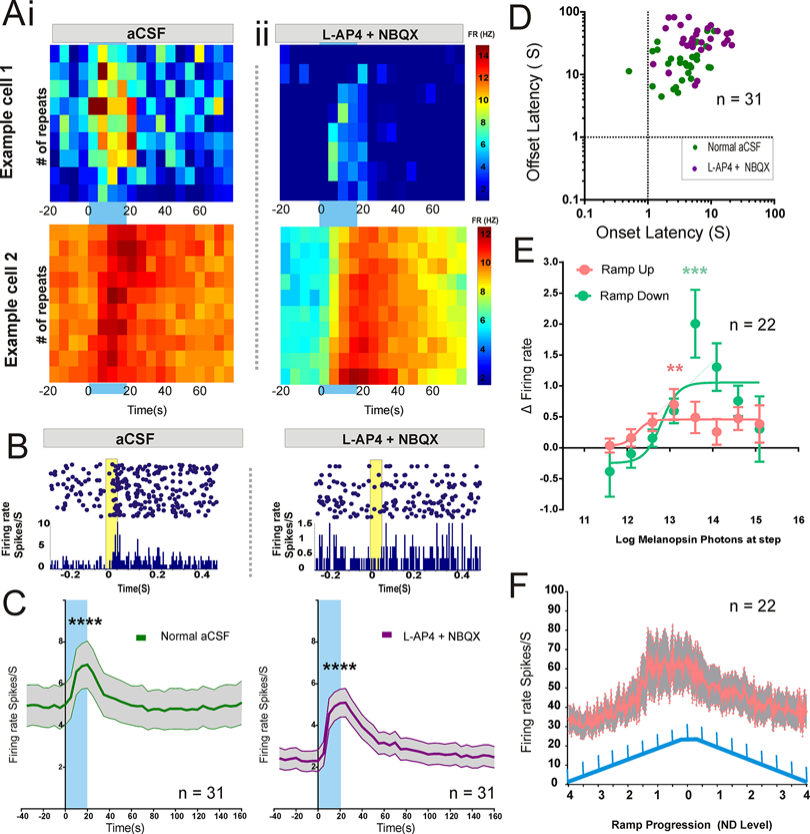
Responses to melanopsin-isolating steps and gradual irradiance ramps in retina. A: Trial bin count examples of two cells responding to a 71% contrast step (blue bar at 0 to 20 seconds) presented during protocol 1 (background log 12.75 melanopsin photons/cm^2^/s to step log 13.5 photons/cm^2^/s) before (i) and during (ii) synaptic blockade. Before and after graphs are scaled to same axis to show changes in baseline activity upon synaptic blockade. B: Example raster plots and PSTH to a 50ms rod favouring yellow flash from dark (flash intensity log 11.45 rod photons/cm^2^/s) under normal conditions (aCSF) and under synaptic blockade (5 minutes after application of L-AP4 + NBQX) in a cell that was identified to respond to our melanopsin-isolating stimulus. C: Averaged plots for firing rate over time of consistent (n = 31) melanopsin-step responsive cells (mean±SEM) before (left) and during (right) synaptic blockade. D: Mean response onset and offset latencies for individual melanopsin-step responsive cells in the presence (purple symbols) and absence (green) of synaptic blockade. E: Under protocol 2, retinal cells responding to a melanopsin-step (n = 22/ 314 cellls; 71% contrast) do so over a range of background irradiances on both the upward and downward phases of the ramp (Repeated measures 2-way ANOVA, step vs baseline firing rate x irradiance; main effects of irradiance (p<0.001), step vs baseline (p<0.001) and interaction (p<0.05); Bonferroni post-hoc comparisons marked on figure as ** and *** p<0.01 for ND2 upward ramp and ND1.5 for downward; n = 22 cells). F: Retinal melanopsin-step responsive cells also tracked the gradual change in irradiance during protocol 2, revealed as a change in firing rate (mean± SEM) as a function of ramp progression.

As our starting point for generating the rod-isoluminant stimuli to be used in the Cgna3^-/-^ mice, we used the known spectral sensitivity function of mouse rod opsin to calculate the irradiance of yellow and blue stimuli that should appear equally bright for rods. We estimated that a switch from the yellow to blue stimulus would represent a contrast of ~71% for melanopsin, matching the blue contrast stimuli we used in *rd/rd cl* animals. In order to confirm that such a stimulus really could elicit melanopsin responses selectively we first recorded extracellular electrophysiological responses from the retina *in vitro*. We found that when held under bright yellow light (12.75 log melanopsin-effective photons/cm^2^/sec), switches to a rod-matched blue (protocol 1; 20s duration; 180s inter-stimulus-interval) induced modest but sustained increases in firing (paired t-test firing rate in baseline vs step p<0.05) in 36/174 light responsive units (see Fig 3Ai for example cells). To confirm that these responses originated with melanopsin photoreception we applied drugs targeting post-synaptic responses to rod activation (L-AP4 and NBQX). This successfully abolished responses to dim 50ms yellow flashes from dark (a rod stimulus; Fig 3B) confirming that rod input was blocked. Under these conditions, clear responses to the melanopsin isolating steps survived this intervention in 31 of 36 units, consistent with the idea that they originated with an intrinsic light response (Fig 3Aii and 3C). There are a number of potential explanations for the loss of responses following pharmacological blockade in the remaining few cells. One is that blue and yellow stimuli were not perfectly rod isoluminant for these cells. However, as their response latency under normal aCSF was typical of that of melanopsin ((n = 5; onset 7.5s +/- 1.2s, range 4.61s-11.22s; offset 30.09s +/- 8.67s, range 8.56s-50.08s), it is more likely that their loss following pharmacological block reflects an impact of this treatment on the melanopsin-driven response (see below) or simply experimental drift. Analysing the firing rate change of these consistent 31 cells with a repeated measures ANOVA revealed both a significant effect of the step (baseline 30 seconds vs step 20 seconds F(1,30) = 66.17, P<0.0001) and also a significant effect of drug condition (F(1,30) = 6.34, P<0.05) seen in the change in baseline firing rate upon the application of synaptic blockers. Both pre and post drug, the difference between the baseline and step firing rates in these 31 cells was highly significant (Fig 3C pre and post drug Bonferroni P<0.0001). Interestingly, the onset and offset latencies in these cells were greater upon application of synaptic blockers (n = 31; pre-drug onset 4.48s +/- 0.49, offset 18.30s +/- 2.09; post drug onset 7.48s +/- 1.03 offset 38.21s +/- 3.56), revealing a sluggish response profile more akin to those observed in *rd/rd cl* mice (students paired t-test between matched pre and post cells n = 31; onsets (t(20) = 3.007, P<0.01; offsets t(30) = 6.101, P<0.0001; Fig 3D).

In a separate group of retinas (n = 8) we examined responses to the melanopsin isolating stimulus step against a gradual ramp in background light intensity (protocol 2). Under these conditions, we found significant differences between baseline and step in around 7% of units (22 cells out of 314 light responsive units) over a range of background irradiances (Fig 3E; static baseline 10 seconds vs 30 second step, students t-test P<0.05). By performing a repeated measures 2-way ANOVA on the firing rates, we found significant effects of melanopsin steps (step vs baseline firing rate; F(1,21) = 22.74, P<0.0001), of background irradiance (across all NDs of ramp; F(16,336) = 9.505, P<0.0001), and an interaction (F(16,336) = 1.95, p<0.05); Bonferroni post-hoc comparisons p<0.01 for ND2 upward ramp and ND1.5 for downward; n = 22 cells). The threshold irradiance for these light adapted step responses was quite high (step irradiance ~ 13.1log melanopsin-effective photons/cm2/s for the upward ramp and 13.6 log melanopsin-effective photons/cm2/s for the downward ramp. In line with the known irradiance-coding properties of ipRGCs we found that the firing rate of those retinal units responding to the melanopsin-steps also tracked the gradual change in background irradiance (Fig 3F). As both protocols 1 and 2 appeared well suited to detect ipRGC function in retina, we moved on to using them to describe melanopsin-derived signals in the dLGN.

### Validation of rod-isoluminant stimuli in dLGN *in vivo*

**Fig 4:**
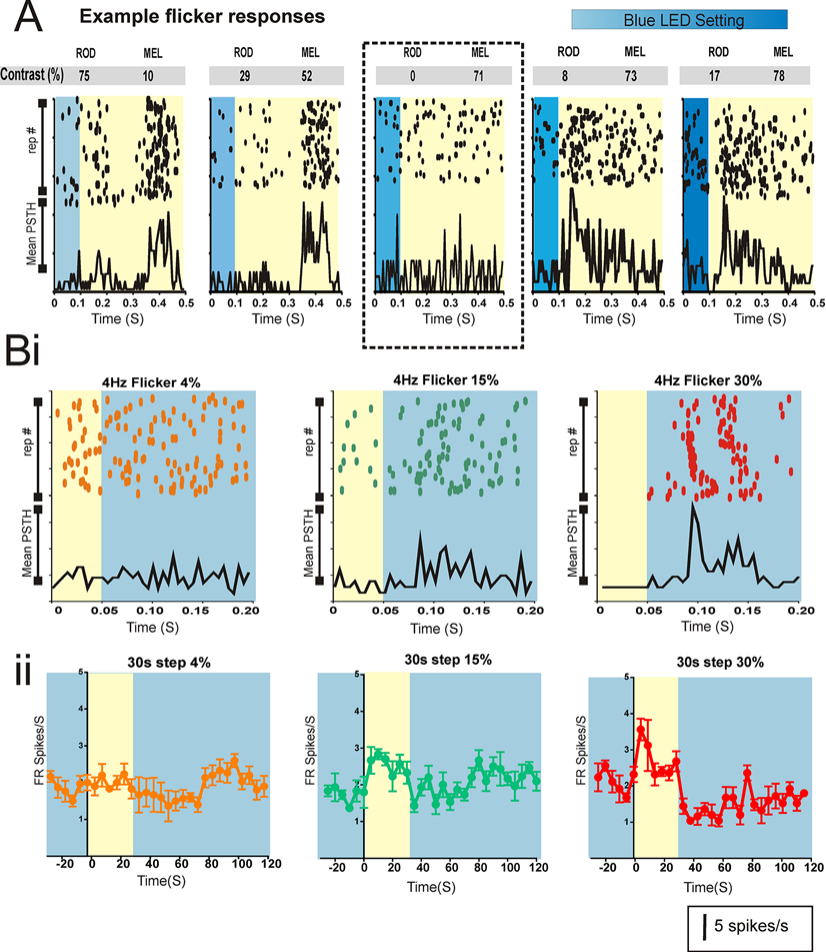
Online calibration and rod control verify silent substitution paradigm. Online calibration of the rod-isoluminant settings (A) and tests of rod contrast sensitivity (B) were performed both *in vitro* and *in vivo*. Displayed are representative data from dLGN. A. A 100 ms blue ‘flash’ (transition from yellow to blue LED) was presented at ND3 to provide conditions preferable to rods. Raster plots and associated PSTHs for an example melanopsin-stepping cell over a range of settings for the blue LED. In the middle (outlined by black dotted box) was the setting at which there was no change in firing, taken as the point of rod isoluminance, while decreasing (plots to left) or increasing (plots to right) the blue LED produced measurable responses in line with the appearance of negative or positive contrast for rods. Numbers above in the grey panels are estimated Michelson contrast for rods and melanopsin calculated according to known pigment absorption nomograms [21]. B: We determined the ability of fast flicker and extended step stimuli to reveal responses to low contrast rod-isolating stimuli (yellow step on low blue background; values above are estimated Michelson contrast). Rod responses were apparent for estimated rod contrasts ≥15% under both a 4Hz flicker (Bi; raster above and PSTH of mean firing rate) and 30sec step (Bii; mean±SEM firing rate) in this representative cell. Firing rates for both A and B match scale bar (bottom left 5 spikes/s).

When applying this strategy to *in vivo* recordings, we first changed our estimate of the irradiance for blue and yellow stimuli required to achieve rod-isoluminance according to estimates of pre-receptoral spectral filtering in the mouse eye [21, 22]. We used these as the starting point for empirically determining the pair of blue/yellow stimuli that were indistinguishable for rods. To this end, we presented transitions between blue and yellow stimuli under conditions predicted to be unfavourable for eliciting a melanopsin response: at moderate light intensity (ND3 11.92 log rod-effective photons/cm2/s) and at 2 or 4Hz flicker (100 or 50 ms blue flash against a yellow background). By modulating the irradiance of the blue LED, we were able to test a range of settings around the point at which we predicted the flash and background to be rod-isoluminant (Fig 4A). We indeed found that there was a setting at which the switch from yellow to blue induced no apparent response (taken as the point of rod isoluminance). However, slight increases or decreases in blue output induced measurable changes in firing confirming the suitability of this method for revealing even fairly modest rod contrasts (Fig 4A). In almost all cases the measured point of rod isoluminance was equivalent to our initial prediction, while in the remaining it was reached with changes in the blue LED output of <4%.

Having confirmed our ability to produce a stimulus pair that failed to elicit measurable rod responses when presented as a flicker, we next set out to confirm that this conclusion was applicable also for the long steps with which we proposed to elicit melanopsin responses. Specifically, we wished to confirm that responses to long light steps were not more sensitive to small rod responses than were those elicited by the fastest flicker (4Hz) used for our online calibration. To this end, we generated separate pairs of stimuli calculated to be isoluminant for melanopsin but presenting Michelson contrasts for rods estimated at 4, 15 and 30%. When presented at 4Hz flicker we were able to measure responses to 15 and 30%, but not 4% rod contrasts, confirming the suitability of this approach for revealing rod contrasts >15% (Fig 4Bi). We then applied 30s steps at a rod contrast of 4, 15 and 30%. Once again, we found measurable responses only to the two higher contrasts (Fig 4Bii), indicating that our dLGN units were no more sensitive to small rod contrasts when presented as long steps than fast flickers, and that our flicker calibration protocol was therefore an adequate method of identifying the point of rod isoluminance.

### Melanopsin driven responses in dLGN under light-adapted conditions

**Fig 5:**
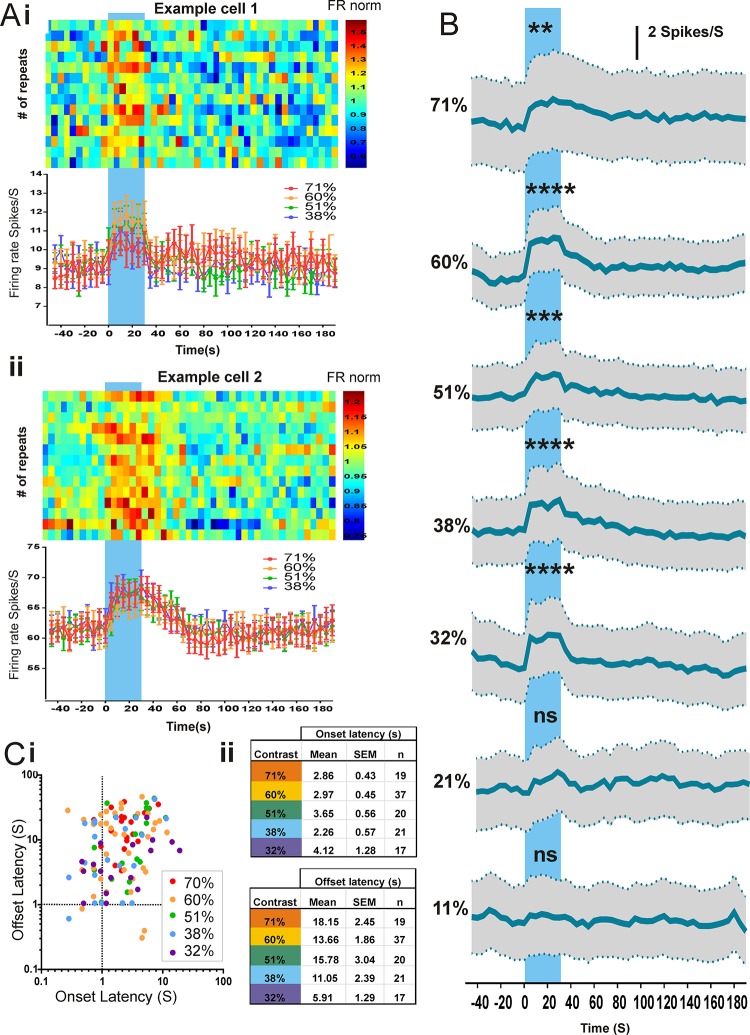
Responses to melanopsin-steps in the dLGN. A: Example cell responses (Ai and ii) to 30s melanopsin-isolating steps (blue shading) at ND0 (background = 14.3 log melanopsin photons/cm^2^/s). Top panels: trial bin count data for multiple repeats of the 71% contrast step (data normalised to mean firing rate of each repeat). Lower panels: PSTHs displaying firing rate over time for 71, 60, 51 and 38% contrast steps (mean±SEM of 14–16 repeats). Bin size in both plots is 5 seconds. B: Average PSTHs (mean±SEM) for all step-responsive cells across a range of melanopsin contrasts. A small but significant change in firing rate can be seen for contrasts ≥32% (two way ANOVA comparing step firing rate vs baseline firing rate (p<0.001), contrast (p>0.05), interaction (p<0.001) with Bonferroni post hoc tests displaying significance level; ** p<0.01; ***p<0.001. C: Mean onset and offset latencies for individual step-responsive units over a range of contrasts. Ci: Scatter plot displaying each single cell response onset vs response offset and ii: mean latencies across all step responsive units.

When the calibrated rod-silent, yellow-to-blue steps were presented under melanopsin-favouring conditions (30s steps against a bright background; 14.31 log melanopsin photons/cm^2^/s), we found measurable changes in firing rate in 54 out of 196 light responsive (LR) dLGN units from 5 animals. Across a range of melanopsin-effective contrasts, the response to these melanopsin-isolating steps comprised a modest increase in firing that persisted throughout the 30sec step duration (2-way repeated measures ANOVA, difference for phase (30 seconds of baseline vs 30 seconds during step) was highly significant; F(1,218) = 112, P<0.0001; Fig 5A and see 5B for Bonferroni t tests). At the highest contrast (71%) we found a few cells that appeared to be inhibited (5 units, data not shown), but as this was never observed at lower contrasts or at dimmer backgrounds this appeared not to be a robust response type. Across those units showing increased firing there was great diversity in response kinetics. The fastest units showed significant increases in firing within a few hundred msec of step onset, with most of the remaining units responding within a few seconds (Fig 5Ci and ii). It typically took >10sec for firing to begin to relax back to basal levels at the end of the step, and in almost every case the response decayed more slowly than it started.

The minimum contrast for eliciting a significant response was surprisingly low (32% Michelson contrast, corresponding to a doubling of effective photon flux). Above this threshold there was no clear relationship between response amplitude and stimulus contrast, with stimuli in the 32–71% contrast range all increasing firing rate by between 1 and 2 Hz on average (2-way repeated measures ANOVA, differences in firing rates across contrasts 71–32% was not significant; F(6,218) = 0.7, P = 0.63; Fig 5B). A subset (7/54) of responsive cells experienced all 7 contrasts, none of these showed a convincing relationship between response amplitude and contrast (data not shown). The lack of a clear contrast: amplitude relationship is surprising. One possible explanation for this is that our inter-stimulus interval was not sufficiently long for melanopin’s sensitivity to fully recover from a previous contrast step. However at 210 sec, it is as long as practicable for a reasonable recording epoch. Onset latency also appeared unrelated to contrast (1 way ANOVA for 71–32% contrast onset latencies (F(4,109) = 1.06, P = 0.38), although it did appear to take longer for firing to return to baseline at the end of the pulse for higher contrast stimuli (1 way ANOVA for offset latencies F(4,109) = 3.35, P<0.05; Significant Bonferroni difference between 71 and 32% only, P,<0.01; see Fig 5Cii for latencies).

### The effect of changes in background irradiance

**Fig 6:**
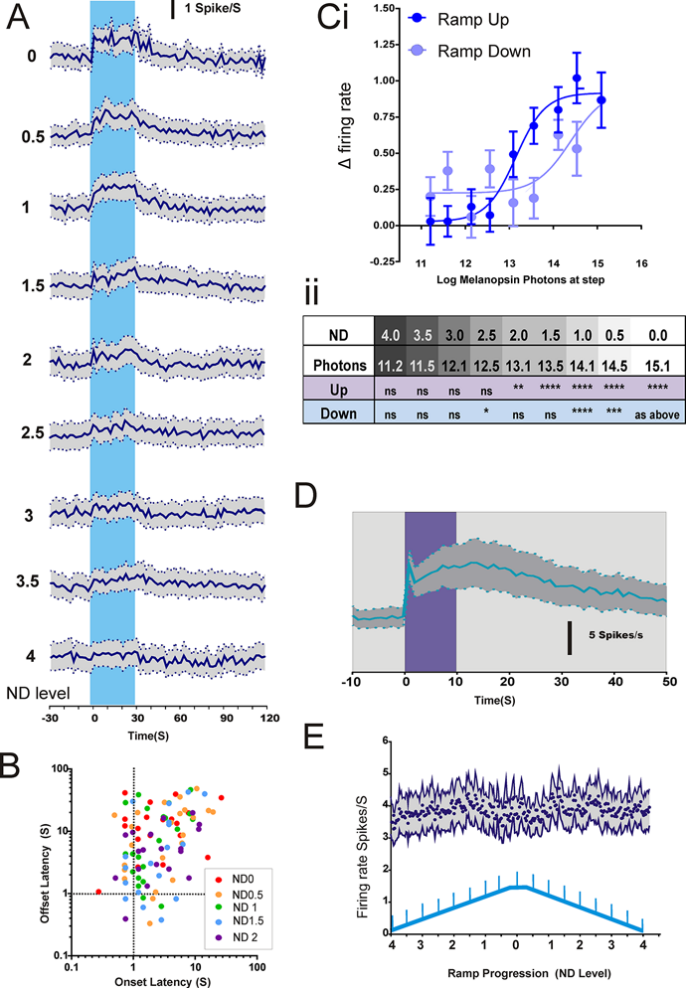
Responses of cells sensitive to the melanopsin-step over a range of irradiances. A: PSTHs for firing rate across melanopsin-isolating steps (71% contrast) over a range of backgrounds (across both increasing and decreasing arms of the ramp; n = 24 responsive cells mean±SEM). See 5Cii for significance values. B: Scatter plot displaying mean onset vs offset latencies for each cell as a function of background irradiance. Ci: Mean change in firing rate associated with the melanopsin-isolating step (firing rate during step—firing rate over previous 10 s) with increasing irradiance during the upwards (dark blue) and downwards (light blue) phases of the ramping protocol. Ci and ii Significant responses were recorded for steps against backgrounds ≥ 12.1 log melanopsin photons/cm^2^/s (ND2) on the ramp up and 13.1 log melanopsin photons/cm^2^/s (ND1) on the ramp down (2-way RM ANOVA step vs baseline firing rage x irradiance; main effects of irradiance (p<0.05), step vs baseline (p<0.001) and interaction (p<0.001); Bonferroni post-hoc comparisons p<0.01 for ND2 and above for upward ramp and ND1 for downward). D: Firing rate (mean±SEM; n = 54) of dLGN stepping cells from protocol 1 to a bright blue 10 sec step from dark (log 14.1 melanopsin photons/cm^2^/s; purple bar). Note sustained activity after the termination of the step, considered to be a feature of the melanopsin light response. E: There was no significant change in time averaged firing rate of cells responsive to the melanopsin step as a function of ramp progression (mean±SEM n = 24).

In order to determine the range of background light intensities over which melanopsin could influence vision, we next recorded responses to our 71% melanopsin contrast step superimposed upon a gradual, spectrally neutral, modulation in irradiance. In this protocol, background light intensity ramped up and down over 4 decimal orders covering the mesopic to high photopic range (bg of 10.48 to 14.29 log melanopsin-effective photons/cm^2^/s; Fig 1D). The melanopsin-isolating step stimulus was presented at half log intervals across this range. Once again, we detected many units (24/189) that responded to the melanopsin step under these conditions (Fig 6A; repeated measures ANOVA revealed a significant effect of baseline vs step firing rate (F(1,23) = 25.90, P<0.0001). At the brighter backgrounds these responses had properties similar to those recorded under the steady background. The kinetics of these responses varied (Fig 6B), with some showing rapid onsets at <1s and others taking >5 sec. However, we did not observe a clear relationship between irradiance and response latency.

The threshold irradiance for detecting these light-adapted melanopsin-driven responses was significantly greater than that reported for either rod or cone photoreception [23, 24]. Thus, we did not record significant changes in firing to backgrounds less than 10^12^ melanopsin photons/cm^2^/sec (ND2, Fig 6A and 6Ci and ii). Interestingly, the sensitivity of this response seemed strongly influenced by light history (repeated measures ANOVA detected an effect of ND (16,368) = 1.86, P<0.05 with a significant interaction between phase and ND fractions (F(16,368) = 6.272, P<0.0001). Although responses to the melanopsin step could be recorded during the ramp down, they appeared only at the higher irradiances, indicating a shift in sensitivity of around 1 log unit compared to the ramp up phase of the experiment (see Fig 6Cii for Bonferroni pairwise comparisons between baseline and step firing rate).

**Fig 7:**
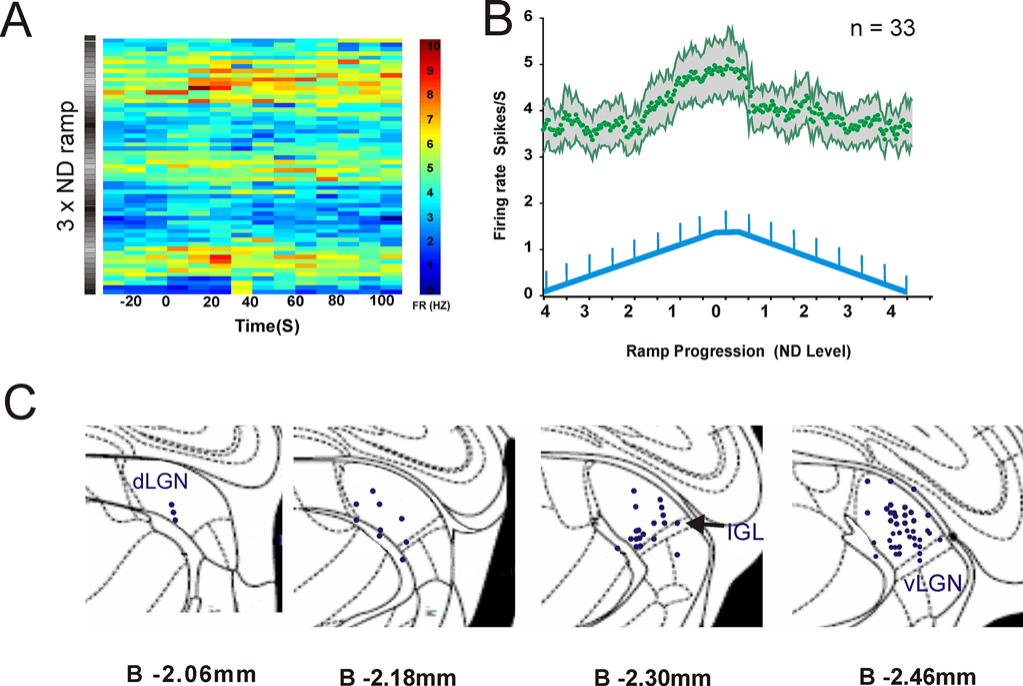
A subset of dLGN cells track the irradiance ramp. A: Trial Bin Count plots from a representative irradiance tracking unit to repeated 130 sec epochs across 3 presentations of the irradiance ramps. Note increases in firing rate in line with the intensity of ND wedge (Black = ND4, white = ND0). B: firing rate (mean±SEM) of 33 units that tracked irradiance (across 3 repeats of the ramp) track irradiance cleanly upwards of ND2 (log 12.1 background melanopsin photons/cm^2^/s). C: Histological localisation of units responding to the melanopsin-isolating steps in protocol 1 or 2 (n = 78; black dots) superimposed upon images from the appropriate elements of a mouse brain atlas showing the boundaries of the dLGN, ventral LGN (vLGN) and neighbouring nuclei (IGL; intergeniculate leaflet).

In common with previous reports of melanopsin responsive cells in the dLGN we found that those units responsive to the melanopsin-isolating step displayed very persistent firing (a fundamental property of the ipRGC light response) when stimulated with a bright blue pulse under dark adapted conditions (6D). On the other hand, given what we found in retina, one surprising aspect of this cell population was that we could not detect a change in the magnitude of cell firing associated with the gradual ramps in background irradiance (Fig 6E). This did not reflect a fundamental inability to resolve such responses *in vivo* as such irradiance tracking was apparent in a separate population of cells in dLGN (33/194 light responsive units). These units tracked increases in irradiance reliably, with firing rate gradually increasing as background irradiance ramped up from 12.29 to 14.29 log melanopsin-effective photons/cm^2^/sec (Fig 7A and 7B). They did not however display any detectable change to the fast melanopsin step at least under these conditions.

### Unit localisation in the dLGN

Reconstruction of recording sites (7C) revealed that cells responding to the melanopsin step were scattered across the lateral geniculate, including both dorsal and ventral components. There appeared to be a tendency for the step-responsive units to be more common in the dorsal LGN, but our sample size was too small to make this distinction with certainty.

## Discussion

This study is one of a small number to examine melanopsin-driven responses under light-adapted conditions [8, 25–27]. Our approach is distinct from previous strategies in that it allows us to isolate melanopsin responses from those driven by both rods and cones without deafferenting ipRGCs through the application of synaptic blockers or using rodless+coneless mice. In order to achieve this, we have had to employ mice lacking cone vision (Cnga3^-/-^). These animals lack the cone-specific cyclic nucleotide gated channel (CNG3), and consequently any cone photoresponse [14]. Cones degenerate gradually over the life of these animals (cone signal remains tonically ‘on’), but rod activity and functional connections from the outer retina to retinal ganglion cells are retained. While it is certainly possible that melanopsin signals are aberrant in this animal, this model more closely approximates the intact visual system than do rodless+coneless preparations. Consistent with this view, we find that melanopsin driven responses have better temporal fidelity and appear more reproducible when elicited by silent substitution in the Cnga3^-/-^ than following pharmacological deafferentation in this model or in the rodless+coneless retina.

An aspect of the silent substitution approach that needs careful consideration is the possibility that the responses we record in fact arise in part because the yellow and blue transitions were not perfectly isoluminant for rods. Several aspects of our data suggest that this is not the case. Importantly, we have not simply predicted which pair of short and longer wavelength stimuli should be rod isoluminant based upon published rod spectral sensitivity, but rather determined empirically the point of effective rod isoluminance for each individual preparation. We show that transitions between this pair fail to elicit measurable changes in firing under rod-favouring conditions with a large number of stimulus repeats. It is therefore most unlikely that the responses we see with many fewer stimulus repeats under conditions favouring melanopsin could arise from rods. Moreover, we see from the *in vitro* preparation that responses elicited by this stimulus are retained following pharmacological deafferentation of retinal ganglion cells. Finally, a couple of aspects of the response to melanopsin-isolating steps are inconsistent with a rod origin. Firstly, such responses appear only at higher background light intensities, when rods should be becoming increasingly saturated, and not at the lower backgrounds when one would expect the system to be more sensitive to small rod contrasts. Secondly, these melanopsin responses have very slow onsets (>several hundred msec) compared to those elicited by rods (Fig 4).

Using this approach to isolate melanopsin-driven responses, we estimate that melanopsin can influence the response to light steps of ~25–30% of dLGN cells. This is a large number considering that ipRGCs comprise <10% of all retinal ganglion cells, but is consistent with previous estimates based upon other approaches [5, 8] suggesting that the influence of ipRGCs extends beyond their single-synaptic targets. When we presented the melanopsin isolating steps against ramps in irradiance we found a somewhat smaller fraction of cells responded (~12%). This likely reflects, at least in part, the difficulty in resolving such responses with so few stimulus repeats. On the other hand, we found an additional ~17% of units whose firing rate tracked the irradiance ramps well. Such irradiance-tracking behaviour was observed for ipRGCs in the retina here and is consistent with previous reports of their sensory characteristics [13, 28, 29]. Thus, while the data presented here do not allow us to resolve this with certainty, it seems likely that the dLGN ramp response could also originate with ipRGCs. Thus, one simple interpretation of our data is that within the dLGN some neurones downstream of ipRGCs respond most robustly to abrupt changes while others more reliably encode background light intensity.

The first question we set out to ask of the responses to melanopsin steps recorded in the dLGN was the range of background light intensities over which they occurred. We find that the lowest irradiance at which we observe a significant response to our melanopsin-isolating step under light-adapted conditions is 10^12^ melanopsin-effective photons/cm^2^/s. This corresponds to twilight levels of ambient light [20] and is similar to the threshold irradiance for eliciting responses from *rd/rd cl* mice to light pulses under dark-adapted conditions [5, 25]. On the other hand, it is relatively high compared to other *in vivo* assays of melanopsin sensitivity, as both circadian and pupil responses have been recorded with stimuli 10-100x dimmer in *rd/rd cl* mice [30, 31]. The brain regions responsible for circadian entrainment and the pupil light reflex receive input from the M1 class of ipRGCs, unlike the dLGN which is thought to receive input from predominantly M4 cells [7, 29, 32]. Given that M1 cells contain more melanopsin and, as such, have enhanced (and more sensitive) intrinsic light-responses, this provides one simple explanation for this difference in absolute sensitivity [7, 32]. Our data further reveal that the threshold irradiance for eliciting melanopsin-driven step responses is strongly impacted by prior light exposure. In the dLGN, we found that when presented during the decreasing irradiance ramp, the melanopsin-step only elicited responses at backgrounds >10^13^ melanopsin-effective photons/cm^2^/s. These data reveal a long lasting light adaptation *in vivo*, equivalent to that reported for ipRGCs *in vitro* [26, 27]. Interestingly, although the protocols applied *in vivo* and *in vitro* were identical, and, in both cases were preceded by a long period of dark adaptation, we did not find an equivalent effect in our *in vitro* retinal experiments. Nevertheless, we cannot exclude the possibility that melanopsin behaves differently in the artificial *in vitro* environment. An alternative explanation is that the melanopsin signal in the dLGN is subject to some central mechanism of light adaptation. This could also explain why units in the dLGN responding reliably to melanopsin steps do not also have a clear response to the slowly changing ramp. This light adaptation could also contribute to the high saturation point for melanopsin step responses—we recorded strong responses to melanopsin steps presented against the brightest background (10^14^ melanopsin-effective photons/cm^2^/s) within the daylight range [20].

Across brighter backgrounds, the amplitude of melanopsin-step responses was uncorrelated with background irradiance, implying that under these conditions responses are defined by the relative, rather than absolute increase in effective photon flux. In other words, these cells can rely upon melanopsin input to encode visual contrast. We explicitly addressed the question of the contrast sensitivity of this melanopsin response at the highest background (10^14^ melanopsin-effective photons/cm^2^/s). This revealed that even a relatively modest increase in light intensity (roughly 2 fold; Michelson contrast 32%) elicited a measurable response. This is well within the variation in radiance found across natural scenes.

These data therefore reveal that a subset of cells in dLGN can use melanopsin to track even fairly modest changes in light intensity over a wide range of background light intensities. An important limitation on the ability of this signal to encode spatiotemporal patterns is its relatively poor temporal resolution. In *rd/rd cl* mice it can take up to tens of seconds for dLGN neurones to respond to light pulses [5]. That was not the case here. Response latency for the melanopsin-step was always long (>100 msec) compared to that expected for rod or cone based signals, but was often <1 sec and with a few exceptions <5 sec. This is consistent with a previous report that the melanopsin-component of the M1 ipRGC is faster under light adapted conditions [27]. These latencies also lie within the realistic range for timescales of visual fixation. It took longer (in most cases >10 sec) for firing rates to begin to return to baseline after the end of the melanopsin-step.

Notably, the latency of the melanopsin response seemed sensitive to outer retinal signalling, as responses to the melanopsin-step in our *in vitro* retinal preparation were slower to onset and more persistent to offset in the presence of synaptic blockers and also in the case of retinal degeneration in our *rd/rd cl* model. This could be related to the reduction in basal firing we observed after applying the synaptic blockers. In the intact retina, ipRGCs receive synaptic input from amacrine and bipolar cells [33–36]. It is presumably some aspect of this circuitry that both improves the temporal fidelity of the melanopsin response and sustains baseline firing. A related question is whether melanopsin responses may be better still in a fully intact retina in which both rods and cones are functional. A particular concern is the sluggish offset kinetics we observe here, as previous recordings from ipRGCs and their targets in the olivary pretectal nucleus indicates that cones play an important role in inhibiting such sustained [37, 38]).

A surprising aspect of our data was that we could not detect a response to the gradual ramps in background irradiance in those dLGN neurones responding to melanopsin-isolating steps. ipRGCs are thought to encode irradiance and scale their firing rate according to steady state light levels over many hours [39]. Accordingly, in the retina, we found that cells responding to melanopsin steps also ramped their firing rate with the background. If we assume that those ipRGCs projecting to the dLGN are represented in this sample, this implies that irradiance and melanopsin-contrast signals are superimposed in the retina.

Turning to the dLGN, our data could suggest two avenues: It is important to remember that because the ramp protocol takes a long time, we are able to include relatively few repeats for this stimulus. As changes in firing induced by both melanopsin-steps and the irradiance ramps are small, this means that it is hard to conclude with certainty that those units with a detectable step response really lacked an irradiance response, or conversely, that the units with an irradiance response really failed to respond to the step. It could be therefore that ipRGC recipient cells in dLGN receive both types of melanopsin-information but our protocol was suboptimal to detect this. On the other hand, under the conditions tested in this study, we found cells with detectable ramp responses and others with detectable step responses with no obvious overlap. Our data therefore indicate, that under our conditions, the dLGN population separates into a group whose response to rapid changes in melanopsin excitation is more detectable than their response to slow changes in background intensity and another group for which the opposite is true. This in turn implies that, at least to some extent, cells in the dLGN extract different sorts of information from the signals provided by ipRGCs. Specifically, some cells likely use the ipRGC input to track slow changes in irradiance and do not respond well to the abrupt melanopsin steps presented here (perhaps because these cells employ a mechanism that reduces their sensitivity to faster changes). Conversely, those cells with a strong response to the melanopsin steps are biased towards responding to more abrupt changes in ipRGC firing and thus appeared insensitive to the background irradiance. As such behaviour implies some sort of central light adaptation mechanism, this can also explain why central responses to melanopsin-steps were inhibited for the downward phase of the irradiance ramp in a way that was not apparent in the retina.

In summary, we find that ~25–30% of neurones in the mouse dLGN are able to use melanopsin to track even relatively modest and quite rapid increases in light intensity. Furthermore, the amplitude of this response is defined by the magnitude of the change in light intensity over a wide range of background irradiances, allowing it to behave as a true response to visual contrast. The melanopsin-derived contrast response has poor temporal fidelity, but in at least a few instances the latency to response is <1 sec, putting it within timescales for visual fixation. These data contribute to defining the conditions under which melanopsin could contribute to the visual system’s ability to detect changes in light intensity over space and time.
